# PPAR Signaling in Placental Development and Function

**DOI:** 10.1155/2008/142082

**Published:** 2007-11-25

**Authors:** Yaacov Barak, Yoel Sadovsky, Tali Shalom-Barak

**Affiliations:** ^1^The Jackson Laboratory, 600 Main Street, Bar Harbor, ME 04609, USA; ^2^Departments of Obstetrics and Gynecology and Cell Biology and Physiology, Washington University School of Medicine, P. O. Box 8064, 4566 Scott Avenue, St. Louis, MO 63110, USA

## Abstract

With the major attention to the pivotal roles of PPARs in diverse aspects of energy metabolism, the essential functions of PPARγ and PPARβ/δ in placental development came as a surprise and were often considered a nuisance en route to their genetic analysis. However, these findings provided an opportune entrée into placental biology. Genetic and pharmacological studies, primarily of knockout animal models and cell culture, uncovered networks of PPARγ and PPARδ, their heterodimeric RXR partners, associated transcriptional coactivators, and target genes, that regulate various aspects of placental development and function. These studies furnish both specific information about trophoblasts and the placenta and potential hints about the functions of PPARs in other tissues and cell types. They reveal that the remarkable versatility of PPARs extends beyond the orchestration of metabolism to the regulation of cellular differentiation, tissue development, and trophoblast-specific functions. This information and its implications are the subject of this review.

## 1. INTRODUCTION

Mammalian reproduction entails prolonged gestation, posing the challenge of securing the thrift and long-term survival of the fetus in utero. The evolutionary answer to this challenge has been the emergence of the placenta, whose roles are to facilitate efficient nutrient, gas and waste exchange between the mother and fetus, while conferring immune privilege on the embryo and secreting pregnancy hormones. The placental core comprises a dense vascular array, where maternal and fetal circulations run in close proximity, but are strictly separated by a trophoblast barrier that specializes in essential bidirectional metabolite transport into and out of the fetus. Placental dysfunction is associated with common disorders of pregnancy, including spontaneous abortions, intrauterine growth restriction (IUGR), and preeclampsia, all of which are commonly associated with compromised placental vasculature [1–3]. In the mouse, dozens of targeted gene mutations result in placental defects that underlie stunted growth or midgestation lethality (reviewed in [4, 5]). Proof of direct causative relationship between such defects and the lethal outcome comes from the complete rescue of embryos by selective reconstitution of the trophoblast in several knockout mouse strains [6–12].

Among the genes whose deficiency results in lethal placental defects are PPARγ and PPARδ; the two are closely related, yet functionally distinct members of the nuclear hormone receptor superfamily of ligand-activated transcription factors. Obligate heterodimers of PPARs and retinoid X receptors (RXRs) bind to PPAR-response elements (PPREs) in the cis-regulatory regions of target genes and activate transcription in response to small lipophilic ligands. While the identities of endogenous PPAR ligands are still inconclusive, pharmaceutical development has yielded several high-affinity synthetic agonists that are widely used in both the clinic and the lab. Importantly, notwithstanding the primary focus of the PPAR field on cellular and systemic metabolism, PPARs and their associated regulators play at least equally essential roles in placental development and function, as reviewed below.

### 1.1. Placental development and trophoblast differentiation

**Figure 1:**
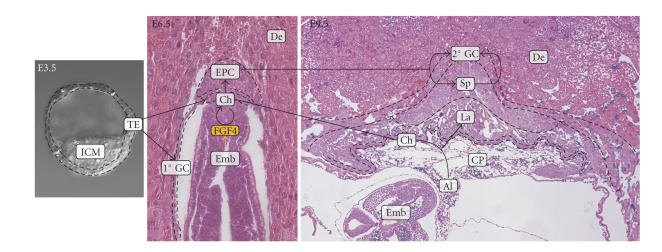
Trophoblast lineages in the developing mouse placenta. Shown from left to right are a blastocyst (E3.5), an E6.5 embryo, and an E9.5 embryo. Respective trophoblast lineages are traced for clarity. Al: allantois; Ch: chrion; CP: chorionic plate; De: decidua; Fmb: embryo; FPC: ectoplacental cone; $1^{{}^{\circ}}\text{GC}$: primary giant cells; $2^{{}^{\circ}}\text{GC}$: secondary giant cells; ICM: inner cell mass; La: labyrinth; Sp: spongiotrophoblast; TF: trophectoderm. FGF4: fibroblast growth factor 4 secreted by the embryo to maintain the chorion. Blastocyst and E6.5 embryo picture courtesy of Drs. Mimi DeVries and Tom Gridley, respectively, The Jackson Laboratory.

The deepest insights into the functions of PPARs in the placenta have been provided by mouse genetic studies. This succinct overview and the accompanying Figure 1 aim at providing the framework for these studies by summarizing placental development in mice. One should bear in mind that while basic principles and molecular regulation of placental development and function are similar across mammals, morphological patterning and architecture of the placenta, and hence terminology, vary considerably among species.

With the exception of the percolating maternal blood, the placenta is exclusively an embryonic tissue. The juxtaposed decidua is a maternal tissue formed from endometrial lining of the uterus. The placenta is comprised of trophoblast cells that originate from the trophectoderm layer of the blastocyst (Figure 1). Implantation of the embryo into the uterine wall triggers the expansion and initial differentiation of trophectoderm cells to form both the chorion and, by process of endoreduplication, primary giant cells. These giant cells facilitate uterine invasion by the embryo. The chorion harbors trophoblast stem cells and, in the mouse, gives rise to the ectoplacental cone (EPC). After initial expansion, the EPC yields the spongiotrophoblast layer and secondary giant cells (Figure 1). Giant cells separate the placenta from the maternal decidua and are responsible both for maintaining the tight placenta-decidua interface and for executing various endocrine functions, including secretion of steroid and prolactin family pregnancy hormones. Spongiotrophoblasts perform (a) endocrine functions by secreting pregnancy specific glycoproteins (PSGs) and prolactin-related hormones, (b) metabolic functions, such as glycogen storage and production of IGF2, and (c) presumed mechanical support functions. Syncytiotrophoblasts that comprise the hemochorial trophoblast barrier between maternal and embryonic circulations (the labyrinthine layer in mice; floating chorionic villi in humans) originate directly from the chorion. In the mouse, vascularization of the placenta initiates around E8.5, when the allantois, which harbors the future umbilical blood vessels, attaches to the chorionic plate. Subsequently, the chorioallantois invaginates into the placenta and lays the vascular framework of the labyrinth. Concomitantly, chorionic trophoblasts in the labyrinth differentiate three morphologically and functionally distinct single cell layers that form a highly specialized epithelial barrier, which execute all bidirectional transport functions between the mother and the fetus. Insights from mouse mutants demonstrate that formations of the labyrinthine trophoblast and placental vascularization are highly concordant and involve extensive cellular and molecular interactions between the allantoic endothelium and the trophoblast [4]. The trophoblast is crucial for placental vascularization, as evident from the complete correction of diverse placental vascular defects by trophoblast-selective rescue [8–12]. In turn, multiple signaling factors secreted by the embryonic endothelium, such as HGF, EGF, LIF, PDGFB, and WNT-2, are essential for proper formation of the labyrinth [13–20].

Cell culture studies have facilitated the mechanistic understanding of molecular and cellular processes involved in various aspects of trophoblast differentiation and function. This area has been markedly advanced by the successful establishment of protocols for procuring and manipulating trophoblast stem (TS) cells from blastocysts or the EPC [21]. The stem cell status of TS cells can be maintained by FGF4 and embryonic fibroblast-derived factors, possibly related to TGFβ or activin [21, 22]. When FGF and conditioned media are withdrawn from the culture medium, mimicking the growing distance between distal trophoblast layers and the embryonic FGF4 source, TS cells differentiate spontaneously, primarily into giant cells and to some extent also into spongiotrophoblast and multinucleated syncytial cells [21, 23]. Moreover, when reintroduced into blastocysts, TS cells are able to undergo differentiate into all trophoblast derivatives [21], demonstrating their true stem cell nature.

## 2. PPARγ

In the absence of prior evidence that PPARγ is expressed during early embryogenesis, the death of *Pparg*-null embryos at the 10th day of gestation (E10.0) was initially surprising [12]. However, further inquiry revealed that *Pparg* is expressed abundantly in the placenta from E8.5 onward, and is not detected in any other embryonic tissue until at least E13.5 (12). This expression pattern provided circumstantial evidence that PPARγ may function in the placenta, but the survival of tetraploid chimeras provided the definitive proof that placental PPARγ deficiency was the cause of embryonic lethality [12]. Tetraploid chimeras are generated by electrofusing 2-cell embryos into single cells with tetraploid genomes. Such embryos resume development, and their aggregation with diploid morulas or embryonic stem cells gives rise to chimeras whose embryo derives exclusively from the diploid partner while their placentas derive from the tetraploid partners [24]. When used to reconstitute diploid *Pparg*-null embryos with WT tetraploid placentas, this procedure allowed survival of the mutant embryos until birth, when they succumbed to unrelated defects that included severe cerebral and intestinal hemorrhages [12]. The recent availability of epiblast-specific Cre transgenes, which delete loxP-flanked (floxed) alleles efficiently in the embryo but not extraembryonic tissue, has enabled to reprove this notion by demonstrating that near-complete deficiency of *Pparg* in the embryo proper is not embryonic lethal [25, 26].

### 2.1. PPARγ and trophoblast differentiation

**Figure 2:**
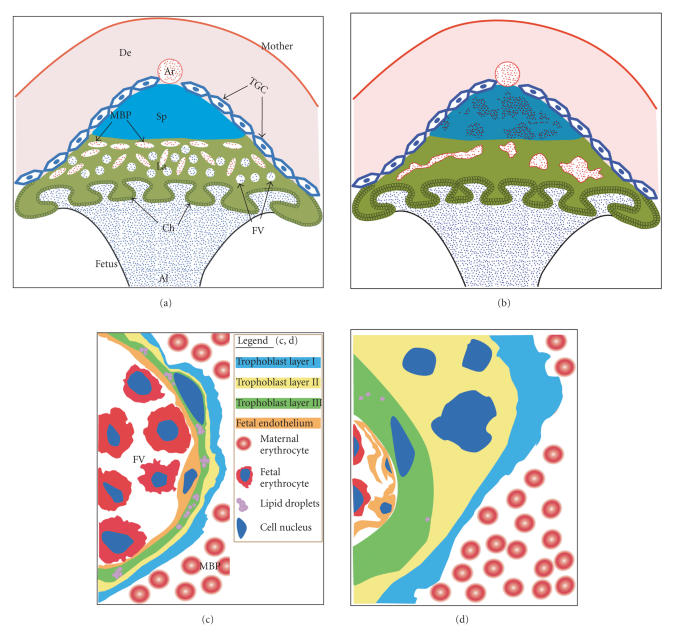
Schematic representation of the *Pparg*-null phenotype. (a) WT placenta. Al: allantois; Ar: maternal artery; Ch: chorion; De: decidua; FV: fetal blood vessels; La: labyrinth; MBP: maternal blood pools; Sp: spongiotrophoblast; TGC: trophoblast giant cells. (b) *Pparg*-null placenta. Corresponding structures are as in (a). Differences of note are marked erythrophagocytosis by spongiotrophoblast cells (red speckles), absence of fetal vessels and breakdown of the maternal blood pools in the labyrinth, and thickening of the chorion. (c,d) Ultrastructural features of WT and *Pparg*-null hemochorial barriers (based on [12]). See legend in (c) for identity of major features. Differences include thickening of the three trophoblast layers, near elimination of lipid droplets in layer III, and loosening of the tight adherence between the trophoblast (green) and fetal endothelium (orange).

The complex histological and ultrastructural phenotype of *Pparg*-null placentas (Figure 2) provided insights into the essential functions of PPARγ. Expression and spatial distribution of prototypic trophoblast lineage markers are intact in the mutant placentas, including the giant cell layer, the spongiotrophoblast, the labyrinth, and the chorion [12]. However, labyrinthine trophoblast precursors fail to terminally undergo differentiate, and instead, retain parenchymal morphology without undergoing either compaction or syncytium formation [12]. The basement membrane between the trophoblast and fetal endothelium is severely disrupted, loosening the critical tight association between the two cell types [12]. This defect likely hampers both the flow of metabolites from the trophoblast to the embryo and the ability of embryonic vessels to use basement membrane tracks for extending and branching into the labyrinth. Consequently, fetal vessels do not permeate the *Pparg*-null placenta and the labyrinthine layer does not effectively form [12]. The trophoblast-lined maternal blood pools are dilated and ruptured, leading to hemorrhages, fibrin deposition, and overt phagocytosis of maternal erythrocytes by junctional zone trophoblasts [12]. Together, these observations indicate that while PPARγ is dispensable for partition of trophoblasts to different lineages, it is essential for terminal differentiation of labyrinthine syncytiotrophoblasts and spongiotrophoblasts, and in turn for placental vascularization and integrity. The further increase of *Pparg* levels in the labyrinth during late gestation suggests that beyond its role in establishing the vascular network of the placenta it may also play an important role in its elaboration and maintenance [27].

On the opposite pole of the PPARγ spectrum, feeding pregnant mice a high dose of the PPARγ agonist rosiglitazone (rosi) from mid to late gestation elicited severe thinning of the spongiotrophoblast layer and substantial dilation of the maternal blood pools in WT placentas [28]. *Pparg^+/-^* placentas were protected from these effects, indicating that these are indeed the result of excessive PPARγ activity. Reduced expression of the trophoblast stem cell marker *Eomes* in rosi-treated WT placentas [28] suggested that excessive PPARγ activity might cause these effects by accelerating stem cell differentiation, concomitantly depleting the stem cell pool and destabilizing the balance between differentiated trophoblast cell types in the placenta. Warnings about embryonic toxicity in rats in the inserts of two commonly prescribed PPARγ agonists, Avandia (rosi) and Actos (pioglitazone), may reflect similar phenomena. In contrast, short-term administration of acute doses of rosi to pregnant rats during midgestation or chronic exposure of pregnant mice to moderate doses of rosi was harmless [29, 30], as were anecdotal incidents in which pregnant women were accidentally exposed to the drug [31, 32].

The functions of PPARγ in trophoblast differentiation have been simulated in several in vitro systems. For example, stimulation of primary human term trophoblasts by PPARγ agonists enhanced their differentiation into multinucleated syncytiotrophoblasts, in agreement with the critical role of PPARγ in syncytium formation in the mouse labyrinth [33]. In TS cells, the association of PPARγ with trophoblast differentiation is manifested in its dramatic induction during transition from the undifferentiated to the differentiated state [34]. This pattern demonstrates that PPARγ is integral to the process of trophoblast differentiation and pinpoints TS cells as an ideal platform for studying the placental functions of PPARγ. On this front, we recently established *Pparg*-null TS cell lines, whose analysis is currently underway [35].

### 2.2. PPARγ and trophoblast metabolism

The established roles of PPARγ in systemic and cellular energy metabolism and the importance of trophoblast metabolism for embryonic development raised the plausible hypothesis that PPARγ might regulate metabolic functions of trophoblasts. This idea was strongly supported by the near-complete absence of lipid droplets from the fetal vessel-proximal trophoblast layer of *Pparg*-null placentas as opposed to their WT counterparts, in which these droplets are abundant [12]. Moreover, PPARγ and RXR agonists synergistically stimulate lipid uptake in both cultured trophoblasts in vitro and whole placentas in vivo [28, 36]. These processes are associated with the upregulation of CD36, FABPpm, fatty acid transport proteins 1 and 4 (*Fatp1, Fatp4*), and the lipid droplet proteins adipophilin, S3-12, and MLDP [28, 36]. Thus, PPARγ is an important regulator of lipid dynamics in trophoblasts.

Hypoxia of trophoblasts due to hypoperfusion of the placental bed is a common complication in human pregnancy. Interestingly, agonist-mediated stimulation of PPARγ protects trophoblasts from an acute, but not a long-term apoptotic response to hypoxia [37]. Potential mechanisms underlying this protective effect include PPARγ-dependent differentiation of cytotrophoblasts to syncytiotrophoblasts, which are more resistant to hypoxic death, or direct inhibition of apoptotic pathways by PPARγ.

### 2.3. Other PPARγ functions in trophoblasts

In addition to the role of PPARγ in trophoblast differentiation and metabolism, it appears to contribute to specialized functions of trophoblasts. One of these unique functions is invasion of the endometrium. The strong coexpression of PPARγ and its obligatory RXRα partner in extravillous cytotrophoblasts at the maternal-fetal interface of human embryos suggested that PPARγ might regulate the invasive functions of trophoblasts. The ability of PPARγ and RXR agonists to inhibit matrigel invasion by both primary and transformed trophoblasts, and the enhancement of invasion by PPARγ and RXR antagonists, supported this hypothesis and implicated PPARγ as a negative regulator of the process [38, 39]. This activity has been correlated to a 3-fold decrease in the expression of pregnancy-associated plasma protein A (PAPP-A)—a protease essential for maturation of the pro-invasive IGF2—and to a 3-fold induction of Interleukin-1β [40].

Another critical function of trophoblasts is the secretion of reproductive hormones, such as placental lactogens (PL) and choriogonadotropin (hCG). Studies in primary human trophoblasts showed that PPARγ and RXR agonists stimulate hCG and hPL production, and that PPARγ-RXRα heterodimers directly activate hCGβ *via* a PPAR-response element (PPRE) in its promoter [33, 38]. These findings suggest that PPARγ functions extend to trophoblast-specific processes beyond cell differentiation, metabolism, and motility.

### 2.4. Placental PPARγ target genes

PPARs are transcription factors, and as such, their raison d’être is to regulate the expression of target genes. Identification of these targets is therefore fundamental for determining the biological functions of PPARs. Two primary philosophies underlie target gene identification. The first is a candidate gene approach, which involves hypothesis-driven testing of genes that make plausible targets based either on their established regulation by PPARs in other tissues or on their known relationship to PPAR-regulated processes; trophoblast targets of PPARs found via this approach are described throughout this review in relation to their biological context. The second approach is discovery-based, and involves unbiased, transcriptome-wide screening for target genes based on genetic, pharmacological, and biochemical criteria. The strength of this strategy lies in its ability to break ground and identify targets whose regulation by PPARs would not be otherwise hypothesized.

The identification of *Muc1* as a PPARγ target gene in trophoblasts by subtraction of cDNA from WT versus *Pparg*-null placentas has proven the power of the latter approach to unearth unexpected targets [34]. *Muc1* is very tightly regulated by PPARγ, and its expression is lost in both *Pparg*-null and *Rxra*-null placentas and is upregulated by PPARγ differentiated TS cells and whole WT placentas [28, 34]. The *Muc1* protein localizes to apical labyrinthine trophoblasts surrounding maternal blood pools, analogous to its luminal localization on simple secretory epithelia, such as those that abut milk or salivary ducts [34]. This spatial pattern invokes unanticipated anatomical and functional analogies between trophoblasts and prototypic luminal epithelia, raising the provocative idea that some of the placental functions of PPARγ are a carryover from more ancient functions in classical epithelia. However, unlike *Pparg*, *Muc1* is not essential for placental development and its deficiency leads at worst to a mild dilation of the maternal blood pools in the labyrinth [34]. This benign phenotype indicates that other target genes must account for the essential placental functions of PPARγ. Our ongoing microarray-based screens start to uncover new PPARγ targets that may account for these functions [35].

In addition to their prospect in illuminating PPAR functions, new target genes provide novel templates for studying the details of native gene regulation by PPARs. Our studies of the *Muc1* promoter provide an excellent example for the unique insights that such an approach can provide over the study of synthetic promoters or isolated response elements. A proximal *Muc1* promoter fragment responds robustly and in an RXRα-dependent manner to PPARγ and rosi, yet unlike most previously studied PPAR targets, let alone synthetic ones, is entirely refractory to PPARα and PPARδ [34]. Detailed mutation analyses reveal a weak PPRE in the proximal part of the *Muc1* promoter that acts as a basal silencer, and whose derepression by PPARγ is required for robust and specific induction of *Muc1* by an upstream, non-PPAR-binding enhancer [34]. This level of detail reveals previously unappreciated layers of specificity and intricacy underlying the regulation of real-life targets by PPARγ.

### 2.5. PPARγ and the placenta-heart axis

Analysis of *Pparg*-null embryos unexpectedly found accelerated cardiomyocyte differentiation and thinning of the ventricular wall [12, 41]. This observation was intriguing because at that developmental stage *Pparg* is expressed nowhere but in the placenta. Consistent with this expression pattern, complete reversal of the cardiac defects in *Pparg*-null tetraploid chimeras confirmed that these anomalies are secondary to the placental defects [12]. This result invoked a previously unappreciated dependence of early heart development on placental integrity [12]. How placental *Pparg* deficiency underlies cardiac malformation is currently unclear and could involve generalized nutritional, vascular, or metabolic deficiencies, hypoxia, or a deficiency for placenta-derived factors. However, similar cardiac defects are often observed in association with placental anomalies (reviewed in [42]), and the “placenta-heart axis” has been since reinforced in *p38a*-null embryos, which phenocopy the *Pparg*-null placental and cardiac defects and are similarly rescued by tetraploid chimeras [11]. Therefore, myocardial failure is likely a general attribute of placental insufficiency and not a specific consequence of PPARγ mutation.

## 3. PPARδ

As in the case of PPARγ, the finding that *Ppard*-null embryos succumb to lethal placental defects was also unexpected [43, 44]. The first *Ppard*-null mouse strain reported was generated by truncating the gene a mere 60 amino acids from its C-terminus (*Ppard*-ΔC60), leaving the entire DNA-binding domain and most of the ligand-binding domain intact [45]. While this allele is likely a hypomorph, the authors reported significantly smaller size and lower survival rates of the original F2 homozygotes for this allele, which they have overcome by outbreeding and consecutive mating of the survivors  [45]. In contrast, mice in which PPARδ was inactivated by CRE/*loxP*-mediated truncation of the N-terminal half of the DNA-binding domain and frame-shifting of the remaining 3’ part of *Ppard* mRNA exhibited overwhelming embryonic lethality and placental defects, as detailed in Section 3.1 [43]. Nevertheless, a few homozygous-null mice survived gestation thanks to a complex influence of genetics and maternal physiology (see Section 3.2). Two other null configurations, one with *lacZ* insertion into the DNA-binding domain of PPARδ [46, 47] and another that replaced the DNA-binding domain with PGK-neo [44], yielded identical lethality and placental defects, confirming that PPARδ is indeed essential for placental function.

### 3.1. PPARδ in placental development and integrity

Lethality and sub-Mendelian ratios of *Ppard*-null embryos are observed from E9.5–10.5 onward. Rare null embryos surviving beyond that stage typically exhibit severe flooding of maternal blood into the placental and embryionic space and are significantly smaller than their WT and heterozygous siblings and the few that survive to birth are markedly runt [43, 44]. Still, none dies after birth and all thrive and become generally healthy and fertile adults, despite remaining slightly smaller than their *Ppard* sufficient counterparts [43]. The combination of strictly prenatal mortality, growth restriction, and abundant expression of *Ppard* in the placenta points to critical defects in extraembryonic tissue.

**Figure 3:**
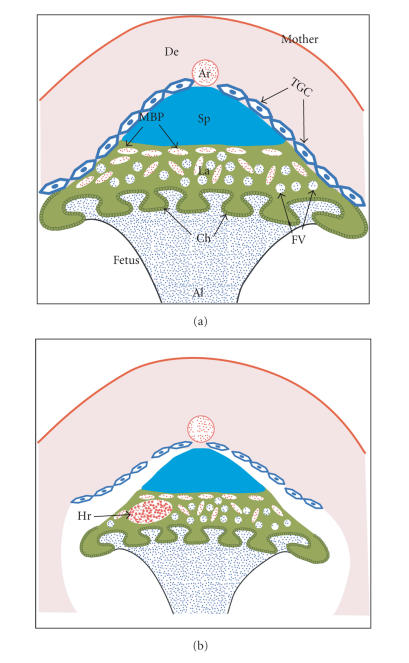
Schematic representation of the *Ppard*-null phenotype. (a) *WT* placenta (similar to Figure 2(a)). (b) *Ppard*-null placenta. Hr: hemorrhage; for all other abbreviations see the legend for Figure 2. Notable differences include smaller and discontinuous giant cells, reduced size of the entire placenta and loosening of its attachment to the decidua, and sporadic severe hemorrhages at various locations in or around the placenta.

From as early as E8.5 onward, *Ppard*-null embryos and placentas are significantly smaller than their littermates [43, 44]. All placental compartments are smaller, including the labyrinth, the spongiotrophoblast, and the giant cell layer. The latter is severely thinner and discontinuous, with cells that do not attain the maximal size typical of WT giant cells (43, 44). This compromise in giant cell size and continuity likely underlies the observed loosening of the normally tight placenta-decidua interface and the inability to retrieve *Ppard*-null specimens from E9.5 onward without substantial detachment of placentas from the deciduas [43]. In contrast, while the labyrinth is smaller, its vascular structure is fully elaborated, clearly distinguishing the *Ppard*-null from the *Pparg*-null placental phenotype [43]. These features are summarized schematically in Figure 3.

Consistent with the implicated role of PPARδ in giant cell differentiationin vivo, studies of the trophoblast cell line Rcho-1 have unequivocally demonstrated that PPARδ is crucial for giant cell differentiation in vitro [44]. Agonist-mediated stimulation of PPARδ dramatically accelerated differentiation of Rcho-1 cells into giant cells, whereas siRNA-mediated knockdown of PPARδ severely inhibited the process. PPARδ was necessary and sufficient for suppression of Id-2, which inhibits giant cell differentiation, and for upregulation of I-mfa, which promotes giant cell differentiation by antagonizing the bHLH transcription factor Mash-2. Interestingly, in trophoblasts, just like in keratinocytes, PPARδ upregulates the expression of two key nodes in the PI3 kinase (PI3K)/Akt signaling pathway: PDK1 and ILK. These, in turn, activate Akt by phosphorylating two residues: Thr308 and Ser473. Activation of this pathway is critical for the ability of PPARδ to accelerate giant cell differentiation, and a synthetic PI3K inhibitor completely reversed upregulation of PL-1, downregulation of Id-2, and giant cell formation. However, additional pathways are at play downstream of PPARδ, as evident in the insensitivity to PI3K inhibition of PPARδ-dependent I-mfa activation.

### 3.2. Genetic and maternal modifications of the Ppard-null phenotype

Surprisingly, all *Ppard* deficient alleles exhibit highly variable penetrance of both the placental phenotype and lethality itself. Our early studies of *Ppard*-null mice encountered a clear maternal effect on the fate of *Ppard*-null embryos. These studies were carried out on either a pure 129/SvJae *129* background or a segregating F2, F3, and F4-C57BL/6J [*B6*]: *129* background, in which the vast majority of homozygous null embryos die during gestation [43]. However, 2–5% of *129*-*Ppard*-null mice and 10–15% of *B6*: *129*-*Ppard*-null mice survived to parturition. These rare survival events were not randomly distributed. First, litters with multiple null pups (up to 4 in one litter) were frequently observed [43, 47]. Second, all survival cases occurred in first-time pregnancies, none recurring in the same breeding pair. Third, survival was not heritable in these cases, that is, null mice were fully fertile, but never gave birth to *Ppard*-null progeny when crossed with *Ppard^+/−^* or *Ppard^−/−^* mates. This substantial deviation from random distribution suggested that survival on these genetic backgrounds is modified primarily by maternal conditions rather than genetics. A hypothetical example of such conditions is slow immune attack of first-time mothers on embryos with breached immune privilege.

Notwithstanding maternal effects, the *Ppard*-null phenotype is also clearly subject to genetic modification. Peters et al. alluded to poor survival of the initial batch of homozygous *Ppard*-ΔC60 mice and the complete resolution of this problem by an additional backcross of F1 mice with inbred C57BL/6N mates, which yielded normal Mendelian distribution of the progeny starting at F3 [45]. Similarly, Nadra et al. reported very low survival rates of outbred *B6:129*-*Ppard*-null mice, which was eventually overcome by intercrossing rare surviving mutants [44]. Our work in progress sheds further light on the effects of genetic modifiers on the *Ppard*-null phenotype. First, repetitive backcrosses onto *B6* completely obliterates survival of mutants beyond E9.5, indicating that *129*-specific alleles allow mutants to survive 1-2 days longer than *B6* alleles and are more permissive towards the survival of *Ppard*-null embryos to term [47]. Second, when *B6:Ppard^+/−^* mice are backcrossed onto an *FVB/NJ* (*FVB*) background, intercrosses of the heterozygous F1 generation result in survival of ∼15% of the expected *Ppard*-null progeny [47]. On this background, survival of F2 *FVB:B6*-*Ppard-null* mice is evenly distributed and not limited to first time pregnancies. Thus, *FVB* alleles are permissive for survival of *Ppard*-null embryos, yet in a substantially different way than the *129* or *B6:129* backgrounds. Third, survival of FVB:B6 *Ppard*-null embryos is heritable, and multigenerational intercrosses of F2-*FVB:B6*-*Ppard*-null parent pairs and their progeny led to the establishment of a semistable stock of viable *Ppard*-null mice [47]. This stock has reached a reproductive plateau by F4, and now consistently yields survival of approximately 50% of the *Ppard*-null progeny. Further inspection reveals that all progeny survive to E10.0, when approximately half of the litter develops abnormal histological features at the placenta-decidua interface and succumbs to transplacental infiltration of maternal blood and fatal hemorrhaging and necrosis. In contrast, the placentas of viable *Ppard*-null embryos from this stock are broadly normal. At present, it is not clear whether this sharp partition represents a stochastically incomplete penetrance or rather a discrete genetic or epigenetic modifier that is inherited by only 50% of the progeny.

In conclusion, placental PPARδ regulates essential processes, which are highly interactive with the genetic and maternal environments. Further studies of the *Ppard*-null phenotype, its response to experimentally defined maternal variables, and identification of genes that modify its nature and outcomes should yield new insights into the biology of both PPARδ and the placenta.

## 4. TRANSCRIPTIONAL PARTNERS OF PPARs

The ability of PPARs to bind DNA and activate transcription depends strictly on heterodimerization with retinoid-X receptors (RXRs) [48]. In addition, diverse transcriptional coactivator proteins are indispensable for transcriptional activation by PPAR-RXR heterodimers. These interdependencies imply that both RXRs and relevant coactivators should be essential for placental functions of PPARs and their deficiencies should yield comparable phenotypes.

### 4.1. RXRs

RXRα is the major RXR isoform in the placenta [49], and its deficiency is therefore expected to recapitulate lethal placental defects of *Pparg*-null and *Ppard*-null embryos. Indeed, *Rxra*-null placentas exhibit multiple defects, some of which are similar to defects in *Pparg*-null placentas, including the following: (a) incomplete compaction of labyrinthine trophoblasts, (b) disruption of the basement membrane and the tight contact between labyrinthine trophoblasts and infiltrating fetal endothelium, (c) a marked reduction in lipid droplet content of labyrinthine trophoblasts, and (d) maternal hematomas at the junctional zone [50]. Other defects, such as partial disorganization of the labyrinthine zone, invasion of spongiotrophoblast cells into the labyrinth, and reduced number of glycogen cells, are not an obvious extrapolation of either the *Pparg*-null or the *Ppard*-null phenotype.

Still, *Rxra*-null embryos die between E12.5 and E16.5 [51, 52], and the aforementioned placental anomalies are observed later than the lethal endpoints of either PPAR deficiency. Therefore, these defects can represent at best an incomplete knockdown of PPARγ and δ activities. This milder phenotype is apparently rooted in functional redundancy with RXRβ, as evident in the markedly accelerated and exacerbated *Rxra*/*Rxrb* double null phenotype [53]. *Rxra*/*b* double null embryos die at E9.5 while exhibiting a combination of failed placental vascularization, which is a hallmark of *Pparg* deficiency, and severe placenta-decidua detachment, as in *Ppard*-null embryos. This phenotype suggests that although RXRα is the primary PPAR partner in the placenta, RXRβ provides a redundant, albeit incomplete backup for PPAR function in the placenta.

The most conspicuous phenotype of *Rxra*-null embryos is severe thinning and incomplete septation of the cardiac ventricles, which is the likely cause of their death [51, 52]. This phenotype is non-cardiomyocyte-autonomous [54] and has been successfully recapitulated by ablation of retinoic acid signaling in the epicardium [55]. Consequently, its relationship to the placental defects has never been investigated. Nevertheless, the proven dependence of myocardial hypoplasia on placental defects in *Pparg*-null embryos raises the need to examine whether at least some aspects of the cardiac *Rxra*-null phenotype can be traced back to placental defects.

### 4.2. CoActivators

Among the large array of cofactors that mediate transactivation functions of PPAR-RXR heterodimers, two stand out in the context of placental functions: PBP/DRIP205/TRAP220 (official gene name: *Pparbp*) and PRIP/AIB3/RAP250 (official name: *Ncoa6*). Three teams knocked out *Pparbp* and found that homozygous null embryos die at E11.5 concomitant with growth restriction and myocardial hypoplasia [56–58]. One team described placental defects that included poor compaction of labyrinthine trophoblasts, reduced vascularization, and phagocytosis of maternal erythrocytes, recapitulating multiple histological and ultrastructural features of *Pparg*-null placentas [56]. These observations suggested that PPARBP coactivates essential developmental targets of PPARγ-RXRα/β heterodimers in the placenta, and the later lethality of these mutants suggested partial redundancy with other coactivators. A second team saw no overt morphological defects in *Pparbp*-null placentas, but found that tetraploid chimeras postponed lethality of the mutants from E11.5 to E13.5, proving that the homozygous-null embryos nevertheless die due to placental defects [57]. Interestingly, tetraploid chimeras did not rescue the cardiac defects of *Pparbp*-null mice, demonstrating that these defects evolve irrespective of the placental problems, unlike in the case of *Pparg* deficiency.

Three teams of investigators generated and analyzed different *Ncoa6*-null mouse strains that exhibited different grades of phenotypic severity [59–61]. One team targeted *Ncoa6* by deleting exons 4 through 7 [59]. Homozygous-null embryos died around E10.0, preceded by substantial growth restriction, severe myocardial thinning, and a series of placental defects that closely resembled those of *Pparg*-null placentas. These included (a) failed vascularization of the labyrinth, (b) poor compaction of syncytiotrophoblasts, (c) dilation and rupture of the maternal blood pools, and (d) erythrophagocytosis in the junctional zone. An additional placental phenotype not shared with *Pparg*-null placentas was thickening of the giant cell layer alongside thinning of the spongiotrophoblast and the labyrinthine zones [59]. These overall similarities indicated that *Ncoa6* is critical for the essential transcriptional functions of PPARγ and perhaps additional transcription factors in the placenta and that *Ncoa6* deficiency is not compensated for by genetic redundancy. The other two teams interrupted the gene downstream of exon 6, and reported undetectable levels of *Ncoa6* gene products, but a significantly milder phenotype [60, 61], which suggested that both configurations are functional hypomorphs. Homozygous-targeted embryos for these alleles died around E13.5 and exhibited myocardial hypoplasia and placental defects that included a thin spongiotrophoblast layer, ectopic spongiotrophoblasts within the labyrinth, reduced vascularization of the labyrinth, and stasis and necrosis in the junctional zone [60, 61]. Interestingly, these features are highly reminiscent of the *Rxra*-null phenotype, suggesting that they indeed reflect incomplete loss of *Pparg* function.

While the phenotypes of *Ncoa6* and *Pparbp*-null mice pinpoint the two as essential coactivators of PPARγ-RXRα/β transcription complexes in the developing placenta, this is by no means the complete inventory of cofactors that are crucial for placental functions of PPARs. First, no cofactor knockout has so far yielded a *Ppard*-null-like phenotype. Second, possible roles of cofactors that have not yielded clear placental phenotypes cannot be ruled out. For example, mice deficient for either CBP or p300 die during early gestation [62–64], and because extraembryonic tissues were not carefully examined in these mutants, placental defects are still a strong possibility. Another complication is presented by families of homologous cofactors with a high potential for functional redundancies, such as the p160 coactivators SRC-1, TIF2, and ACTR/SRC-3 or the PGC-1 family, that is, PGC-1α, PGC-1β, and PRC. While single deficiencies for any of these cofactors are not embryonic lethal, therefore precluding serious placental defects, one should keep in mind that compensation by remaining family members may well be at play.

## 5. CONCLUSIONS AND PROSPECTS

As detailed in this review, PPARγ and PPARδ play nonredundant roles in placental development and physiology. PPARγ is a key regulator of trophoblast differentiation and metabolism, PPARδ is essential for giant cell function and placental integrity, and their coreceptors RXRα and β are instrumental for the execution of these functions. At least two transcriptional coactivators, PPARBP and NCOA6, are critical for essential functions of PPARγ in the placenta, as deduced from the *Pparg*-null-like phenotype of their deficiencies, and additional cofactors are likely crucial for those of PPARδ.

Still, the network of signals upstream, alongside, and downstream of PPARγ and PPARδ is far from elucidated. Several PPAR targets have been identified in trophoblasts, providing initial mechanistic insights into PPAR function in the placenta. However, the discovery of as many new target genes will be indispensable for fully deciphering these functions. Another important effort should be to determine the various regulators that control or modify PPAR expression and activity in trophoblasts. These include, but are not limited to upstream transcriptional regulators, molecules that control the stability of PPAR gene products, posttranslational modifications that alter the functions of PPARs, RXRs, or their cofactors, and the production and dissemination of endogenous ligands. Many of these processes may constitute key regulatory nodes in placental physiology. In addition, PPAR-specific features, such as the identity of genes that modify the outcomes of PPARδ deficiency, would provide invaluable insights.

Finally, identifying compelling similarities between the *Ppar*-null placental phenotypes and published descriptions of targeted genes with previously unknown connections presents a complementary approach for identifying critical nodes in placental PPAR signaling. Such a strategy has been widely successful in identifying a plethora of epistatic relationships in lower eukaryotes such as yeast, nematodes, and flies, and more recently in identifying novel SHH signaling components in mice [65]. Because placental defects are among the earliest roadblocks in the development of many gene-targeted embryos, such opportunities abound. For example, the published analyses of single and compound keratin 8 (*mK8*), *mK18*, and *mK19* knockouts reveal remarkable similarities to the *Ppard*-null placental phenotype [66–69]. Similarly, the placental and cardiac phenotypes of αV- and β8-integrins, p38α, *JunB*, and *Fra1* knockouts are strikingly similar to those of *Pparg*-null embryos [9–11, 70, 71]. Integrating studies of these genes and their corresponding pathways into the functional studies of PPARs and their regulators, associated factors, and transcriptional targets should provide further insights into the mode by which PPAR signaling networks regulate placental development.
